# Homogeneous polyporus polysaccharide inhibits bladder cancer by polarizing macrophages to M1 subtype in tumor microenvironment

**DOI:** 10.1186/s12906-021-03318-x

**Published:** 2021-05-25

**Authors:** Wenyu Jia, Siwan Luo, Gena Lai, Shiqi Li, Shuai Huo, Meifang Li, Xing Zeng

**Affiliations:** 1 The Second Affiliated Hospital, Guangzhou University of Chinese Medicine, Guangzhou, 510120 Guangdong Province China; 2 Guangdong Provincial Hospital of Chinese Medicine, Guangzhou, 510120 Guangdong Province China; 3 Fuwai Central China Cardiovascular Hospital, Zhengzhou, 451464 Henan Province China

**Keywords:** Bladder cancer, Homogeneous polyporus polysaccharide, THP-1-derived macrophage, Tumor microenvironment, JAK2/NF-κB pathway

## Abstract

**Background:**

Polyporus polysaccharide (PPS), an active ingredient of traditional Chinese medicinal Polyporus umbellatus, has multiple biological functions, such as anti-cancer, immune-regulating and hepatoprotective activities. The purpose of this study was to investigate the mechanism of homogeneous polyporus polysaccharide (HPP) activated macrophages in the treatment of bladder cancer.

**Methods:**

100 ng/mL Phorbol myristate acetate (PMA) was used to induce THP-1 human leukemic cells as a macrophage model. Then macrophages derived from THP-1 were treated with different concentrations of HPP (1, 10 and 100 μg/mL). Flow cytometry and RT-PCR were used to detected the expression of CD16, CD23, CD86, CD40 and interleukin (IL)-Iβ, iNOS mRNA. ELISA was used to test the change of IL-1β and TNF-α in macrophage after the treatment with HPP. The conditioned medium from HPP-polarized macrophages was used to detect the effect of activated macrophages on bladder cancer. MTT assay, 5-ethynyl-2′-deoxyuridine assay, flow cytometry, Transwell assay, and Western blot analysis were used to detect the effects of polarized macrophages on the viability, proliferation, apoptosis, and migration of bladder cancer cells. Western blot was also used to analysis the change of JAK2/NF-κB pathway protein.

**Results:**

HPP promoted the expression of pro-inflammatory factors, such as IL-Iβ, TNF-α and iNOS, and surface molecules CD86, CD16, CD23, and CD40 in macrophages and then polarized macrophages to M1 type. Results demonstrated that activated macrophages inhibited the proliferation of bladder cancer cells, regulated their apoptosis, and inhibited migration and epithelial–mesenchymal transformation (EMT). JAK2/NF-κB pathways were downregulated in the anti-bladder cancer process of activated macrophages.

**Conclusion:**

The findings indicated that HPP inhibited the proliferation and progression of bladder cancer by the polarization of macrophages to M1 type, and JAK2/NF-κB pathway was downregulated in the process of anti-bladder cancer.

**Supplementary Information:**

The online version contains supplementary material available at 10.1186/s12906-021-03318-x.

## Background

Bladder cancer is the leading cause of cancer-related deaths in urinary system. Surgical treatment, chemotherapy, and radiotherapy are the main clinical therapeutic regimens for bladder cancer, but the high recurrence rate (60–70%) and mortality are still difficult issues to resolve [1]. Therefore, the necessity of exploring prospective methods to improve the therapeutic effect on bladder cancer is emphasized.

The role of the tumor microenvironment (TME) in tumorigenesis and development is further recognized in solid tumors. Immune cells, including T cells, B cells, NK cells, macrophages, dendritic cells, and neutrophils, in the TME [2] can promote the deterioration of tumors, increase the invasiveness of tumors, and evade host immune response and anti-treatment response. However, under certain conditions, they can be involved in host immune surveillance and killing in tumor [3]. Anti-inflammatory type-2 (M2 macrophage) can promote the tumorigenesis, development, metastasis, and drug resistance of solid tumors, including bladder cancer [4–6], while pro-inflammatory type-1 (M1 macrophage) in turn inhibits these effects [7–9]. Increasing the proportion of M1-subtype cells in TME is a promising strategy for cancer treatment [10, 11]. However, the role of M1-like macrophages polarized by plant polysaccharides in bladder cancer remains unclear.

*P. umbellatus*, belonging to Polyporaceae, has been traditionally used for treatment of edema, acute nephritis and diarrhea (The State Pharmacopoeia Commission of PR China, 2010). Polysaccharide extracted from *P. umbellatus* is the main ingredient of the medicine. Previous studies found that polyporus polysaccharide (PPS) could alleviate the adverse reactions of Bacille Calmette-Guerin (BCG) and improve its efficiency [12], and effectively inhibit the progression of *N*-butyl-*N*-(4-hydroxybutyl)-nitrosamine (BBN)-induced bladder cancer in rats [13]. In addition, PPS was found to increase the expression level of CD86 and CD40 in bladder cancer tissue of rats [12], and regulate the immune activity of macrophages and promoted them to M1-like macrophages [14]. In our previous study, a new polysaccharide (HPP) purified from PPS has been identified and has the activity of polarizing macrophages to M1 subtype. Therefore, it was speculated that HPP could inhibit the progression of bladder cancer by regulating macrophage polarization in tumor macroenvironment. The present study aimed to explore the role of HPP-induced macrophages in bladder cancer cells and its potential molecular mechanism.

## Methods

### Materials and chemicals

The RPMI 1640 medium, fetal bovine serum (FBS), and phosphate-buffered saline (PBS) were procured from Hyclone. Lipopolysaccharide (LPS, From *E. coli*, Cat no: L-2630) was procured from Sigma (MO, USA). Primers for real-time reverse transcriptase–polymerase chain reaction (RT-PCR) and TRIzol (Cat no: 15596026) were purchased from Invitrogen. Revert Aid First-Strand cDNA Synthesis Kit (Cat no: k1621) and FastStart Universal SYBR Green Master (ROX) (Cat no: 4385610) were purchased from Roche. RIPA (Cat no: 9806S) and primary antibodies for Western blot analysis, including anti-IκB (Cat no: 76041S), anti-phospho-IκB (Ser32) (Cat no: 2859S), anti-NF-κB p65(Cat no: 8242S), anti- phospho-NF-κB p65(Ser536) (Cat no: 3033) anti-JAK2(Cat no: 3230S), anti-phospho-JAK2(Tyr1007/1008) (Cat no: 3771S), and anti-GAPDH (Cat no: 5174S), were purchased from Cell Signaling Technology. Secondary antibodies were obtained from Sigma. The Enhanced Chemiluminescence Solution Kit (Cat no: WBKLS0500) was obtained from Millipore (MA, USA). APC mouse anti-human CD16(Cat no: ab203883) and FITC mouse anti-human CD86(Cat no: ab213044) were purchased from Abcam (Cambs, UK). APC mouse anti-human CD40(Cat no: 555591) and FITC mouse anti-human CD23(Cat no: 561146) were purchased from BD Systems (BD Biosciences, USA).

### Preparation of HPP

The preparation process of HPP is described in reference [15]. Its average molecular weight was showed to be 6.88 kDa by HPGPC analysis. In addition, GC and PMP derivatization HPLC analysis determined that HPP was composed only of glucose.

### Culture of THP-1 and differentiation into macrophages

The THP-1 cells were purchased from ATCC and maintained in the RPMI-1640 medium containing 10% FBS, 100 UI/mL penicillin, and 100 μg/mL streptomycin at 37 °C in a humidified incubator with 5% CO_2_. The THP-1 cells (5 × 10^5^ cells/mL) were exposed to 100 ng/mL phorbol myristate acetate (PMA) for 48 h and then cultured for 24 h in the absence of PMA as a recovery period to differentiate into macrophages.

### Analysis of THP-1 membrane surface molecules by flow cytometry

Macrophage-like cells were treated with HPP (1, 10, and 100 μg/mL) or LPS 1 μg/mL for 72 h (LPS-induced M1 macrophages in the positive control group). The THP-1 cells were collected in a flow tube and washed with 2 mL of PBS three times. After removing the supernatant, the cells were incubated with corresponding antibodies for 30 min at 4 °C and protected from light, following analysis with flow cytometry. The expression levels of CD16, CD23, CD40, and CD86 were detected for evaluating the effects of HPP and LPS on THP-1-derived macrophages.

### Detection of target gene mRNA by RT-PCR

THP-1-derived macrophages were treated with HPP (1, 10, and 100 μg/mL) or LPS 1 μg/mL for 6 h, and then the cells were collected for RNA extraction. Total RNA was extracted from THP-1 using TRIzol reagent and converted into cDNA using the Revert Aid First-Strand cDNA Synthesis Kit. PCR was performed according to the FastStart Universal SYBR Green Master. The genes and corresponding primer sequences (forward and reverse) are listed in Table 1.

**Table 1:**
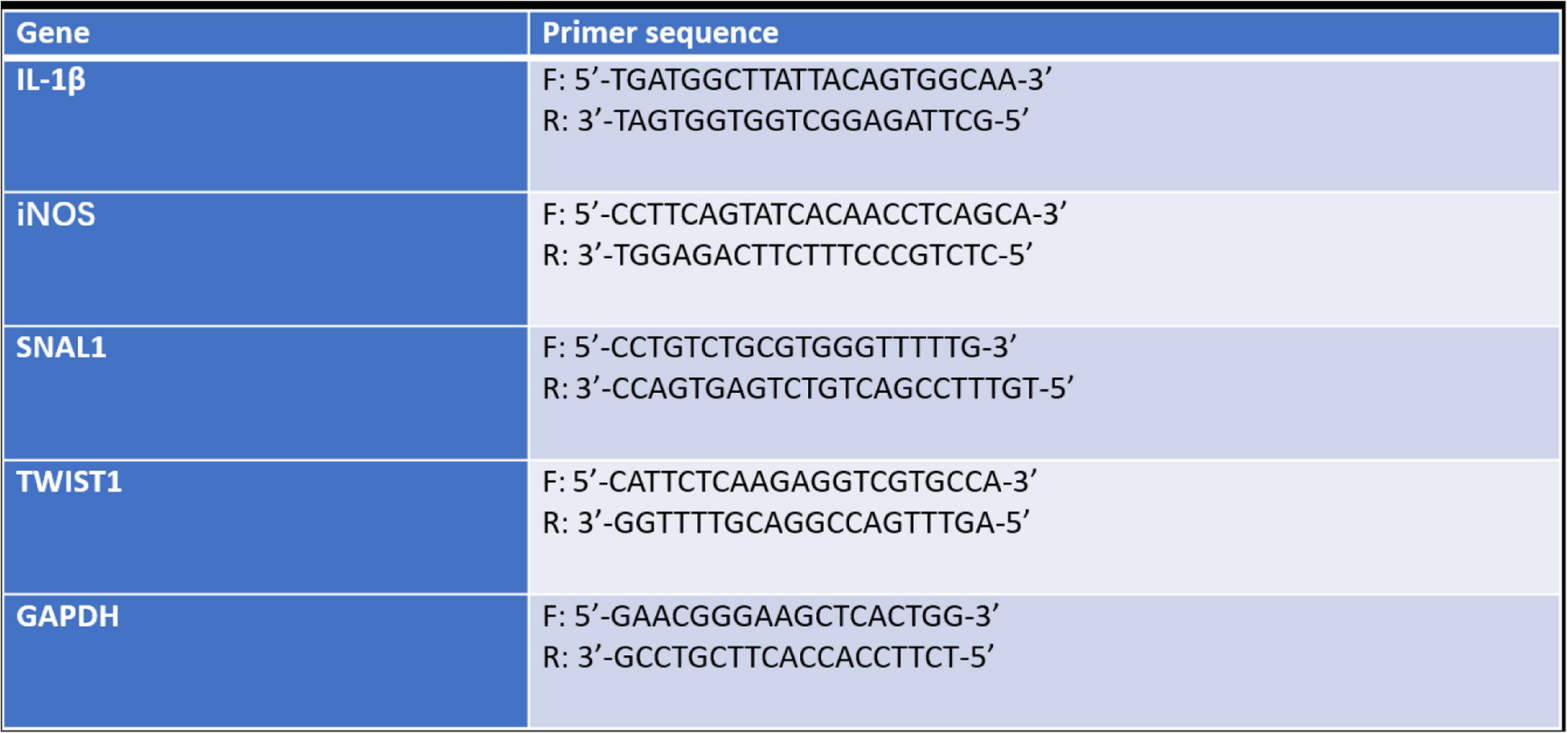
Primer sequences for PCR amplification

### Measurement of expression level of cytokine IL-1βand TNF-α by enzyme-linked immunosorbent assay

After being treated with HPP or LPS, the THP-1 cells in six-well plates at a density of 5 × 10^5^ cells/well were cultured in a fresh medium for 24 h. Cell supernatants were collected and stored at − 80 °C after centrifugation at 4 °C and 350 *g*. The expression levels of cytokines were analyzed using an enzyme-linked immunosorbent assay (ELISA) kit (Abcam). The sample was diluted 20 times before measurement so that the measured absorbance was within the range of the standard curve. The absorption at 450 nm was measured by enzyme labeling to calculate the concentration of cytokines in the sample.

### Preparation of THP-1 cell culture medium and grouping of human bladder cancer cells

The macrophages were incubated with a complete medium containing different concentrations of HPP (1, 10, and 100 μg/mL) or LPS 1 μg/mL for 72 h, and the medium was refreshed for another 24 h. The cell culture supernatant was collected and stored at − 80 °C after centrifugation at 4 °C and 350 *g*. Human bladder cancer cells T24 and EJ were incubated with the THP-1 culture medium and a fresh medium 1:1 for 24 h or 48 h, respectively, which were used in the following experiment.

### Cell proliferation detection using 3-(4,5-dimethyl-2-thiazolyl)-2,5-diphenyl-2-H-tetrazolium bromide assay and 5-ethynyl-2′-deoxyuridine assay

The 3-(4,5-dimethyl-2-thiazolyl)-2,5-diphenyl-2-H-tetrazolium bromide (MTT) analysis kit (Cat no: 0210222701, MPBIO) and 5-ethynyl-2′-deoxyuridine (EdU) staining assay (Cat no: C10311–2, RIBOBIO) were used to assess cell viability and proliferative capacity. T24 and EJ cells (6 × 10^3^ cells per well) were seeded into a 96-well plate, and 6 parallel wells were set up for each group. Following a 24 h incubation, the cells were incubated with the collected conditioned medium. Then, 20 μL of MTT (5 mg/mL) was added to each well and incubated at 37 °C for 4 h. The medium was discarded, and 200-μL of dimethyl sulfoxide was added to each well. The 96-well plate was gently shaken for 10 min. Then, the absorbance was measured at 490 nm. The Cell-Light EdU Apollo 567 In Vitro Kit (RIBOBIO, Guangzhou, China) was used for EdU assay after the cells were cultured with the supernatant of macrophages treated with HPP or LPS in a 96-well plate. After Apollo and Hoechst fluorescence staining, photographs were taken under an inverted fluorescence microscope (Nikon, Tokyo, Japan), and the number of proliferating cells was counted.

### Apoptosis assay

The T24 and EJ cells were seeded in six-well plates at a density of 2.5 × 10^5^ cells/well and incubated with the supernatant for 24 h. The cells were collected, washed with PBS, resuspended in 300 μL of 1× banding buffer, mixed with 5 μL of Annexin V-FITC to avoid light, and incubated at room temperature for 15 min. Then, 5 μL of PI staining was added and incubated at room temperature for 5 min. Apoptosis was detected using flow cytometry. In addition, apoptosis-associated protein markers were detected using Western blot analysis.

### Assessment of protein level using Western blot analysis

The expression of EMT-related marker, and JAK2/NF-κB signaling pathway protein was detected using Western blot analysis. The cells were split using RIPA lysate containing inhibitors of proteases and phosphorylated proteases. Subsequently, the protein was collected and quantified by bicinchoninic acid assay. Then, 8–12% SDS-PAGE was used for protein electrophoresis and then transferred to an Immobilon-P membrane (Millipore). The membranes were saturated with a blocking solution (5% bovine serum albumin) for 1.5 h at room temperature and incubated with a primary antibody overnight at 4 °C. The immunoblot was washed five times with TBST, 5 min each time, and incubated with a secondary antibody for 2 h, which were detected using ECL.

### Cell migration assay

The Transwell migration assay was used to evaluate the metastatic ability of cancer cells. The Transwell chamber had a 6.5-mm diameter insert with an 8.0-μm pore size. The cells treated with the supernatant were starved for 2 h with a serum-free medium. The lower chamber was filled with 600-μL of RPMI 1640 medium containing 10% FBS. The aforementioned treated T24 and EJ cells were counted and resuspended in a serum-free medium, and 200-μL of the cell suspension was added to the upper chamber at a density of 2 × 10^5^/mL. The cells were incubated at 37 °C for 24 h. The cells penetrating the upper chamber were immobilized with methanol for 30 min and stained with crystal violet for 20 min. The cells were photographed under a microscope, and the cells passing through the upper chamber were counted.

### Statistical analysis

The data were presented as a mean ± SD of three independent experimental results. One-way analysis of variance was used for inter-group data. GraphPad Prism 6.0 was used for data processing. A *P* value < 0.05 was considered statistically significant.

## Results

### HPP increased the expression levels of inflammatory factors iNOS and IL-1β and surface molecules CD86, CD16, CD23, and CD40 in macrophages

**Fig. 1:**
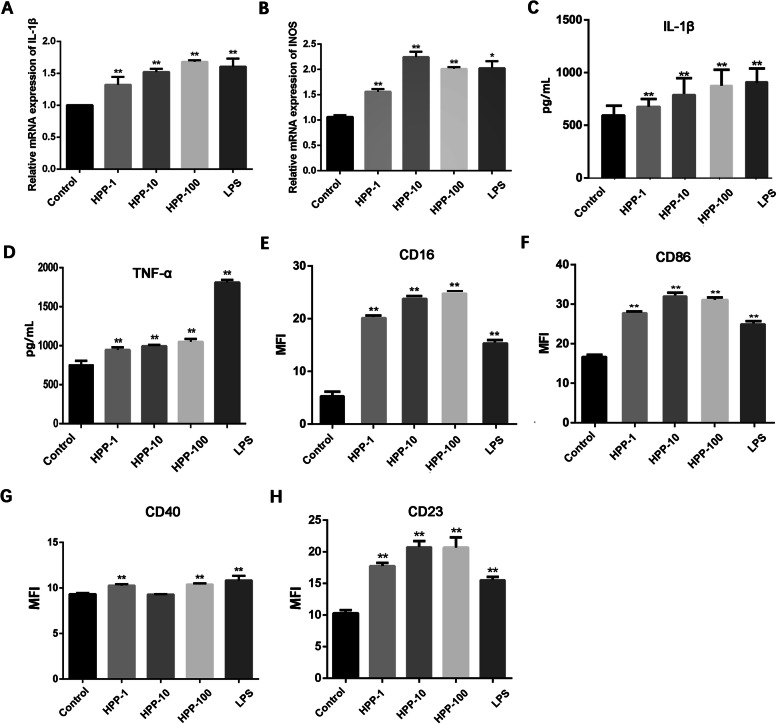
HPP increased the expression levels of inflammatory cytokines, such as IL-1β and NO, secreted by macrophages and promoted the expression of surface molecules CD86 and CD16. **a**-**b** mRNA expression of IL-1β, and iNOS in macrophages after 6 h of HPP or LPS incubation. **c**-**d** HPP or LPS induced IL-1β and TNF-α production in the cell supernatant and the expression levels cytokines were analyzed by ELISA. **e**-**h** THP-1-derived macrophages were pretreated with HPP or LPS for 72 h, then the expression levels of CD23, CD40, CD16, and CD86 were measured by flow cytometry. ^*^P < 0.05; ^**^P < 0.01 compared with the control group(*n* = 3)

The polysaccharides extracted from herbal medicines can promote the production of pro-inflammatory factors by macrophages with their good immunoregulatory activity [16–18]. Compared with the control group, HPP increased the mRNA expression levels of IL-1β, and iNOS (Fig. 1a-b). Furthermore, the ELISA kit was used to detect the change in cytokine levels in the cell supernatant, which demonstrated a significant increase in the expression level of IL-1β and TNF-α (Fig. 1c-d). CD86 and CD16 were phenotypic molecules produced by M1 macrophages [19, 20]. The results showed that both HPP and LPS could promote the expression of M1-type membrane molecules CD16, CD23, CD40, and CD86 on the cell surface (Fig. 1e-h).

### Macrophage incubated with HPP inhibited the viability and proliferation of human bladder cancer cells

**Fig. 2:**
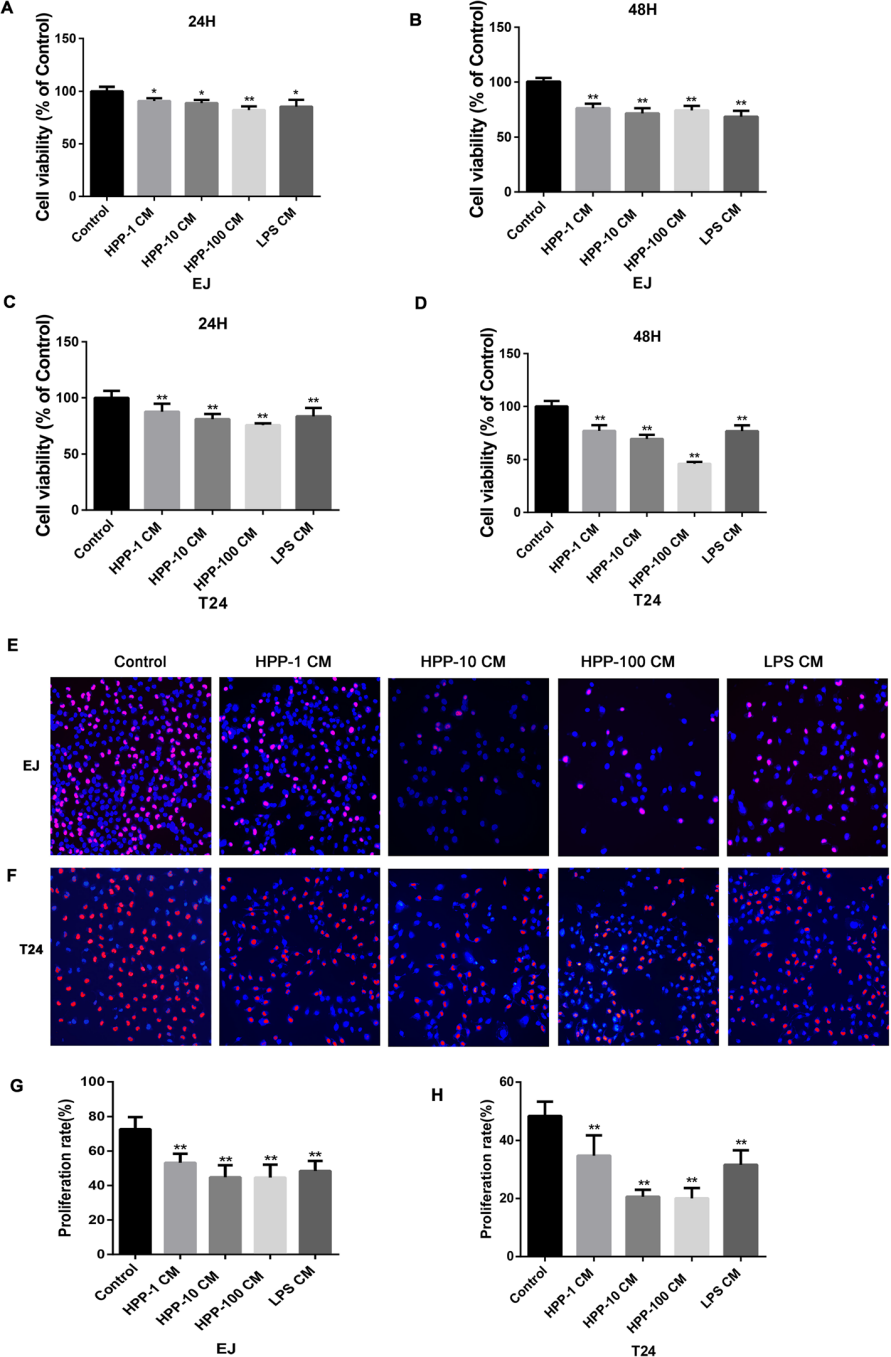
Cell viability and proliferation were restrained when incubated with the aforementioned supernatant. **a**-**d** Change in the cell viability (% of the control group) when human bladder cancer cells T24 and EJ were cultured with the supernatant for 24 h or 48 h. **(e-h)** Effect of activated macrophages on T24 and EJ cell proliferation observed using EdU staining (200×). Red fluorescence denotes EdU staining, representing proliferating cells. Blue fluorescence denotes DAPI staining, representing the staining of nuclei. Statistical data analysis showed a downward trend in cell proliferation. ^*^P < 0.05; ^**^P < 0.01 compared with the control group(n = 3)

The MTT assay was used to detect the changes in the viability of T24 and EJ cells incubated with the conditioned medium at different time points. The optical density (OD) values measured in the treatment group showed a downregulated trend compared with the control group. Figure 2 (a-d) shows that the cell viability of the experimental group on T24 and EJ cells decreased with the prolongation of culture time. In view of the time-dependent inhibitory effect of the conditioned medium on the viability of bladder cancer cells, the cell proliferation was detected 48 h later. An EdU nucleoside should always be used to evaluate cell proliferation [21]. Figure 2 (e-h) shows that the macrophages activated by HPP attenuated the hyperplasia of T24 and EJ cells in a concentration-dependent manner.

### Apoptosis of bladder cancer was enhanced by the HPP-polarized macrophages

**Fig. 3:**
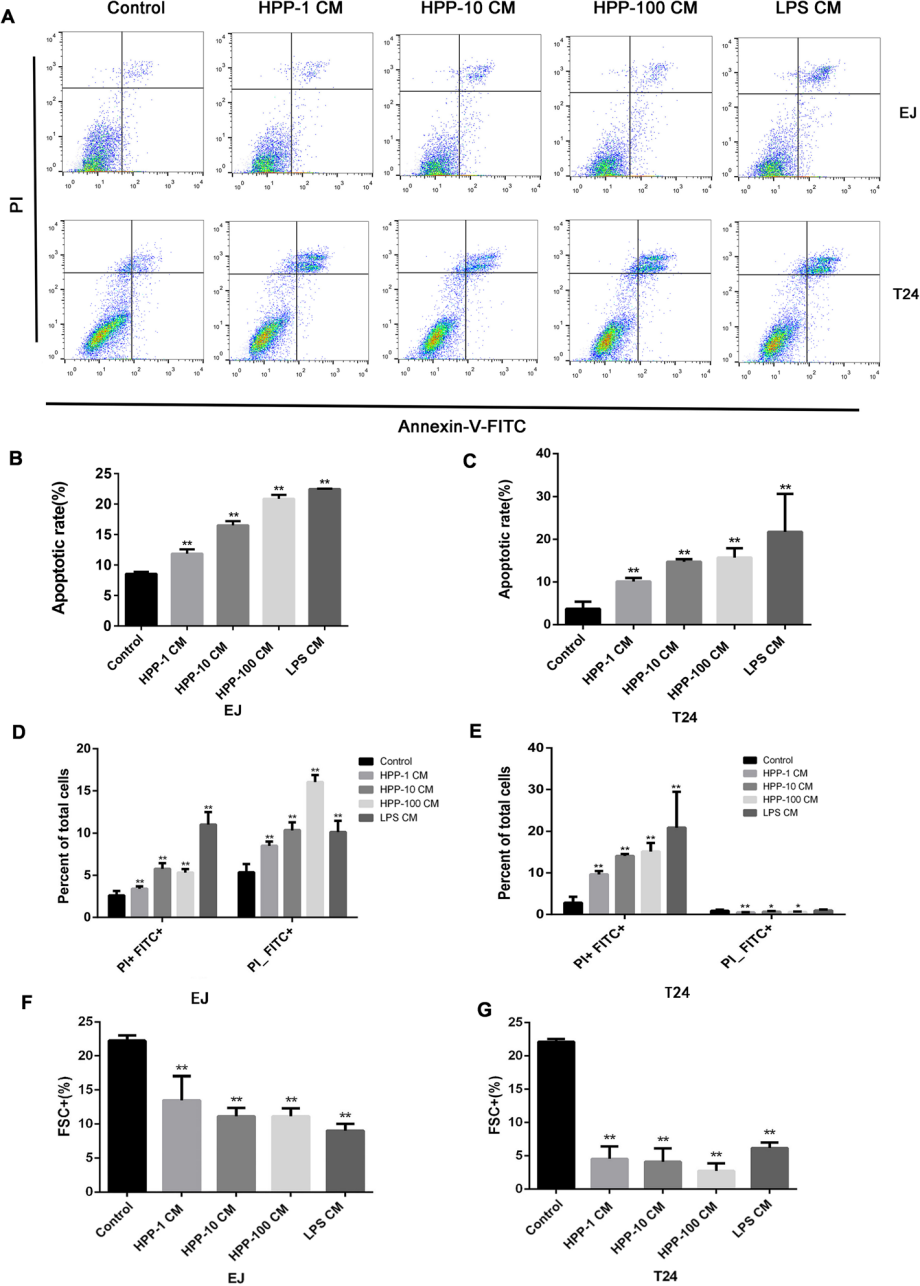
Detection of apoptosis by Annexin V/PI staining. **a-c** HPP-polarized macrophages facilitated the apoptosis of bladder cancer compared with the control group. **d-e** Statistical analysis showed the changes in early and late apoptosis of bladder cancer cell lines T24 and EJ in experimental groups compared with the control group. **f-g** Experimental group showed decreased cell volume using flow cytometry. These experiments were repeated three times for statistics. ^*^P < 0.05, ^**^P < 0.01 compared with the control group(n = 3)

The M1-like macrophages induce apoptosis in cancer cells [22]. Our results showed that HPP-polarized macrophages increased apoptosis obviously compared with the control group, as shown in Fig. 3a. Especially, activated macrophages significantly increased the apoptosis of bladder cancer cells in the late stage rather than in the early stage (Fig. 3d-e). Apoptosis is characterized by nucleocytoplasmic collapse and cell volume reduction. Flow cytometry showed that the cell volume in the experimental group decreased significantly in the present study (Fig. 3f-g).

### Cell migration and epithelial–mesenchymal transformation ability of bladder cancer cells were regulated

**Fig. 4:**
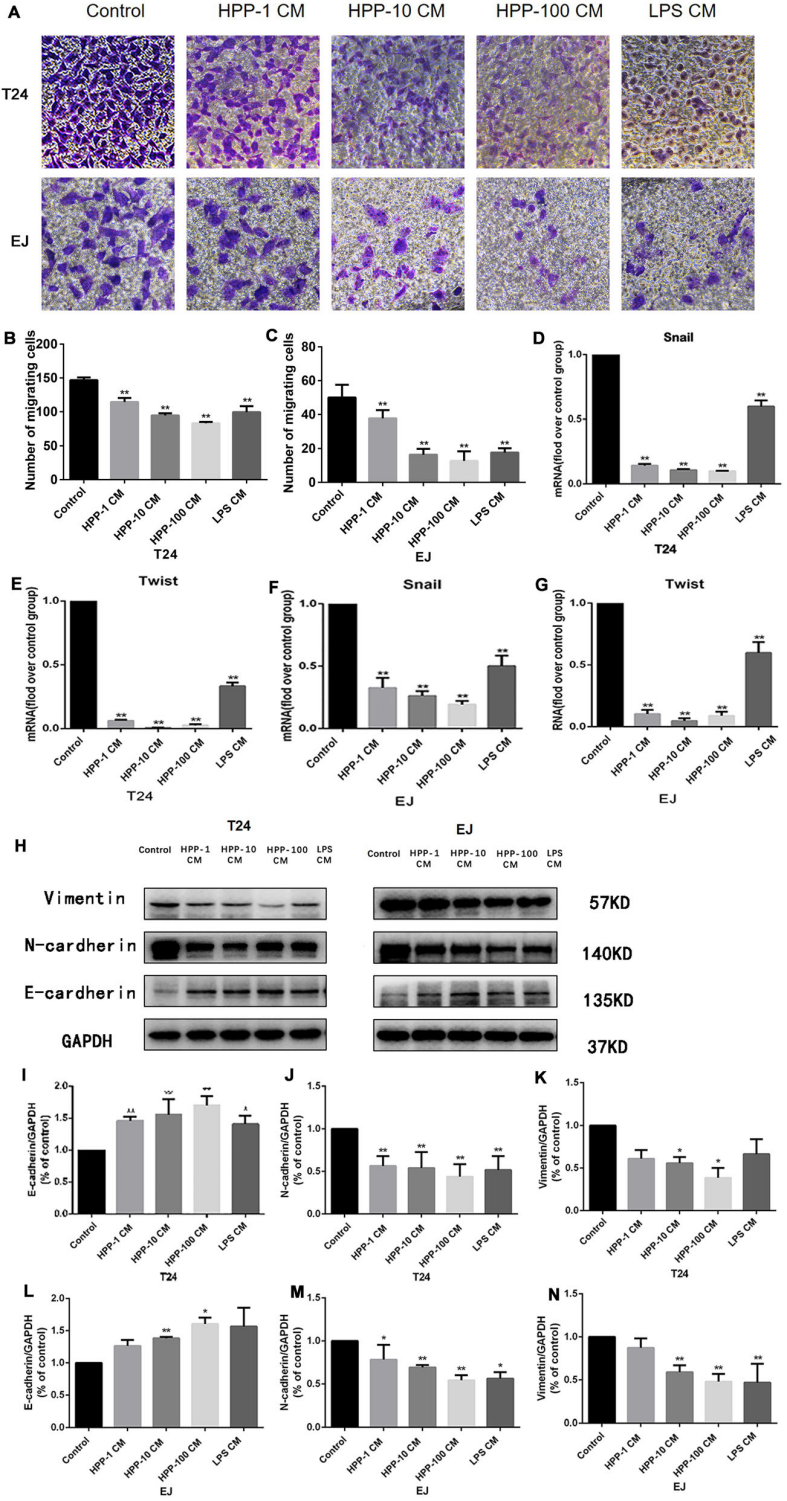
Determination of cell migration ability and epithelial–mesenchymal transition–related proteins using Transwell chamber and Western blot analysis. **a** Microscopic photography of cells penetrating the upper chamber and statistical analysis of migrating cells in different treatment groups (400×). **d-g** RT-PCR analysis of expression of EMT transcription factors. **h-n** Western blot analysis of the expression of EMT marker proteins. ^*^P < 0.05, ^**^P < 0.01 compared with the control group(n = 3)

The ability of cell migration is closely related to the metastasis of bladder cancer. Figure 4a-c shows that the number of cells penetrating the upper chamber decreased significantly after being treated with the cell supernatant of HPP-polarized macrophages, demonstrating that the migration capacity of T24 and EJ cells was inhibited. A close correlation exists between epithelial–mesenchymal transformation and metastasis and invasion of tumors [23]. A conditioned medium of macrophages attenuated the cell migration of bladder cancer cells. Then, the effect of the medium on EMT of bladder cancer was investigated. The expression levels of mesenchymal marker/transcription factors N-cadherin, Snail, Twist, and vimentin in the experimental group significantly decreased, while that of epithelial marker E-cadherin was reversed (Fig. 4d-n).

### JAK2/NF-κB signaling pathway was involved in the inhibitory effect of HPP-polarized macrophages on bladder cancer cells

**Fig. 5:**
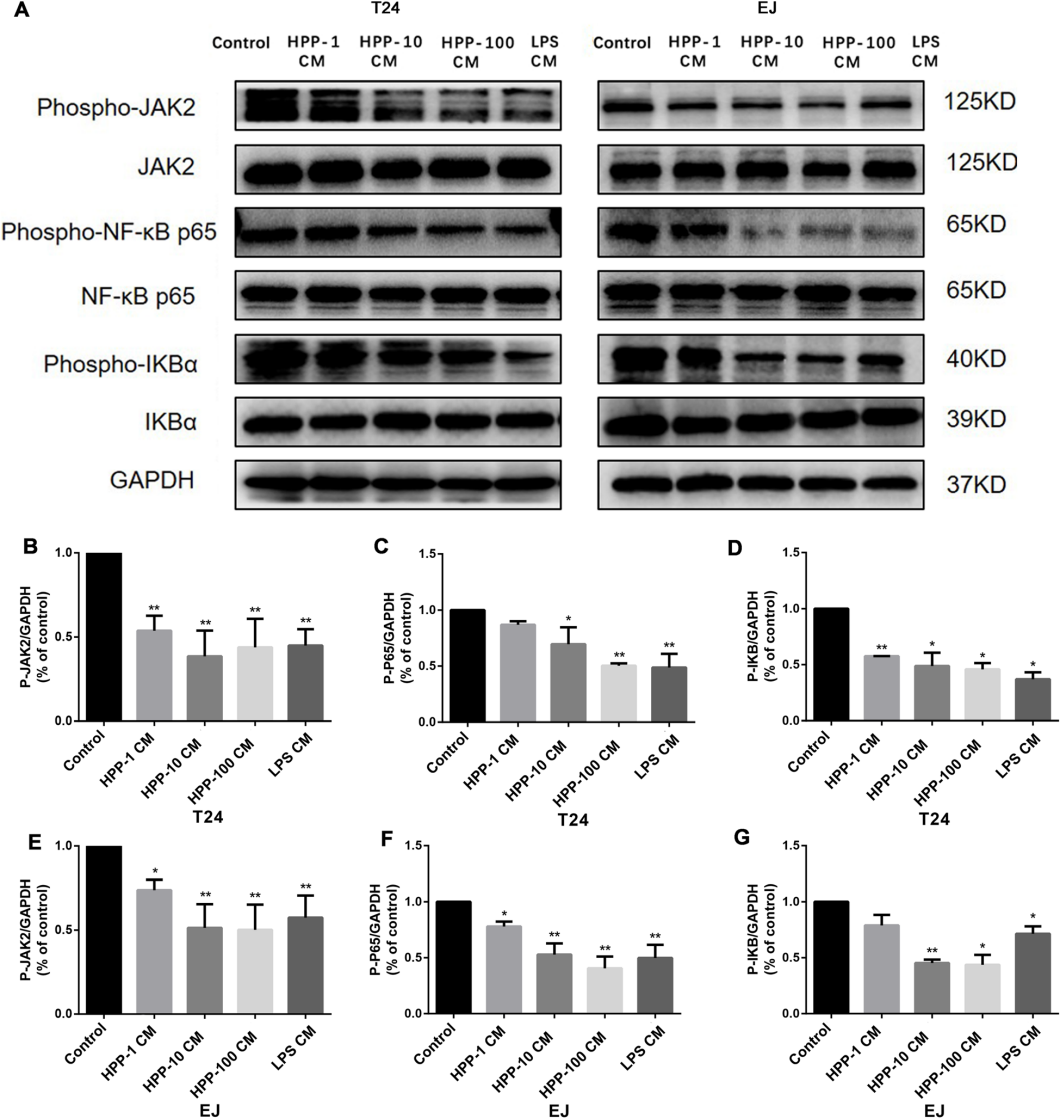
Detection of the expression of JAK2/NF-κB pathway protein by Western blot analysis. The protein levels of p-JAK2, p-P65, and p-IKB were downregulated in bladder cancer cells T24 and EJ after culture with the conditioned medium. ^*^P < 0.05, ^**^P < 0.01 compared with the control group(n = 3)

The JAK2 receptor was reported to be closely related to a series of cell functions, such as cell cycle, apoptosis, genetic stability, and tumor cell migration [24, 25]. The expression of the JAK2/NF-κB signaling pathway protein was examined to explore the potential molecular mechanism of M1-like macrophages regulating the proliferation, apoptosis, migration, and epithelial–mesenchymal transformation of T24 and EJ cells. The Western blot analysis showed that the level of phospho-JAK2 and phospho-NF-κB p65 in T24 and EJ cells decreased after incubation with the conditioned medium (Fig. 5). These data indicated that HPP-induced macrophages regulated the proliferation, apoptosis, migration, and epithelial–mesenchymal transformation of bladder cancer cells through the JAK2/NF-κB pathway.

## Discussion

The relationship between tumor cells and immune cells in TME has become a hot topic in recent years. A large number of clinical and experimental data have confirmed that TAMs can promote the genesis and progression of tumors [5]. In view of the role of macrophages in tumors, several anti-cancer strategies targeting macrophages are available [10]. This study demonstrated that HPP played an anti-bladder cancer role by polarizing macrophage to M1 type. Moreover, it also provided evidence that the downregulation of JAK2/NF-κB pathways was involved in the anti-tumor effect of polarized macrophages.

The M1 macrophages produce pro-inflammatory factors (TNF-α, IL-1β, and iNOS), chemokines (CXCL10, CXCL11, and CCL2), antigen-presenting molecules such as MHCII, costimulatory molecules (CD86 and CD80), and antigen-treated peptidases, which play an anti-tumor role in cancer. The M2 macrophages produce nutritional polyamines, anti-inflammatory factors (IL-10 and TGF-β), and chemokines (CXCL18 and CXCL22), which play a tumorigenic role in cancer [26, 27]. Therefore, seeking drugs that can induce macrophages to become M1 macrophages and then play an anti-tumor role is of great significance.

Plant polysaccharides can induce macrophages to polarize to pro-inflammatory subtypes due to their good immunomodulatory effects [28], indicating that polysaccharides regulate TME and have anti-tumor activity. The aqueous extract of *P. umbellatus* Fries was found to effectively inhibit bladder cancer, and polysaccharides were the main component responsible for its effect; however, little is known about its anti-bladder cancer mechanism. The follow-up studies showed that PPS enhanced the activity of macrophages stimulated by IFN-γ [14]. In addition, PPS had an inhibitory effect on the progression of bladder cancer in BBN-rat models and increased the effect on the expression levels of CD86 and CD40 in peritoneal macrophages [12]. HPP is a homogeneous polysaccharide extracted from PPS, which has been proved to have regulatory activity on macrophages by Toll-like receptor 2 (TLR2) and activated the signaling pathways of NF-κB and NLRP3 [15]. It was speculated that HPP might play an anti-tumor role by regulating the changes in macrophages in the TME of bladder cancer. In this study, in vitro experiments were conducted by simulating the effect of macrophages on tumor cells in TME. PMA-induced THP-1 is a commonly used human macrophage model, which was used in this study. Subsequently, the present study showed that HPP could promote macrophages to secrete inflammatory factors, such as IL-1β and TNF-α; increase the expression levels of membrane surface molecules CD86, CD16, CD23, and CD40; and induce macrophages to differentiate into M1 subtype. The anti-tumor effect of M1 macrophages was confirmed. LPS is the classical inducer of M1-subtype macrophages, which were used as a positive control in this study. IL-1β and TNF-α have anti-tumor effects and can inhibit the growth and metastasis of tumors in vitro and in vivo [29, 30]. CD40 and CD86 were costimulatory molecules on the surface of macrophages. The increased expression levels of CD40 and CD86 can promote T-cell activation and play an anti-tumor role [31]. Next, the conditioned medium of THP-1 was used in this study to explore the effects of HPP-polarized macrophages on bladder cancer cells. As expected, HPP-induced M1-like macrophages inhibited many pathophysiological characteristics of bladder cancer, including proliferation, migration ability, and epithelial–mesenchymal transformation accompanied by increased apoptosis of bladder cancer cells. At the same time, the sensitivity of T24 and EJ cells to activated macrophages was found to be different in this study, which might be caused by different genotypes and cell receptors. Furthermore, the underlying molecular mechanism of its anti-tumor activity was clarified in the present study. Abnormal activation of JAK2 and NF-κB pathways is known to associate inflammation with tumors and is closely related to the malignancy and poor prognosis of tumors [32–34]. The downregulation of JAK2 and NF-κB pathways can regulate the proliferation, apoptosis, and metastasis of tumors [35, 36]. In this study, the downregulation of the JAK2/NF-κB pathway was found to be closely related to the anti-cancer effects of HPP-induced polarized macrophages on bladder cancer. These findings strongly indicated that the homogeneous polyporus polysaccharide could play an anti-bladder cancer role by regulating macrophage polarization to M1 subtype.

## Conclusion

These findings indicated that HPP could inhibit the growth and progression of bladder cancer cells by polarizing THP-1-derived macrophages to M1 type. The downregulation of JAK2/NF-κB pathways might mediate the anti-cancer process.

## Supplementary Information

**Additional file 1: Supplementary Figure 1**. The effect of JAK2 inhibitor (AZD1480) on T24 cells. p-JAK2, p-P65, and p-IKB were downregulated when T24 cells were treated with AZD1480. ^*^P < 0.05, ^**^P < 0.01 compared with the control group(*n* = 3).

**Additional file 2: Supplementary Figure 2**. The effect of HPP-treated macrophage on normal bladder epithelial cells (SV-HUC-1). The conditioned mediums from HPP-treated macrophage have no effect on normal cells while LPS-activated macrophage is not. ^*^P < 0.05, ^**^P < 0.01 compared with the control group(n = 3).

**Additional file 3: Supplementary Figure 3**. The effect of JAK2 inhibitor (AZD1480) on bladder cancer cells. AZD1480 inhibited the cell viability and induced apoptosis of bladder cancer cells. ^*^P < 0.05, ^**^P < 0.01 compared with the control group(n = 3).

## Data Availability

The datasets used in the current study are available from the corresponding author on reasonable request.

## Abbreviations

*P. umbellatus*: Polyporus umbellatus
PPS: polyporus polysaccharide
HPP: homogeneous polyporus polysaccharide
IL-1β: interleukin (IL)-Iβ
PMA: Phorbol myristate acetate
TNF-α: tumor necrosis factor alpha
MTT: 3-(4,5-dimethyl-2-thiazolyl)-2,5-diphenyl-2-H-tetrazolium bromide
EDU: 5-ethynyl-2′-deoxyuridine
EMT: epithelial–mesenchymal transformation
