# Supplementary figures and images for: Homogeneous polyporus polysaccharide inhibits bladder cancer by polarizing macrophages to M1 subtype in tumor microenvironment
Source: BMC Complement Med Ther. 2021 May 25;21:150. doi: 10.1186/s12906-021-03318-x (PMC8152148; doi:10.1186/s12906-021-03318-x)

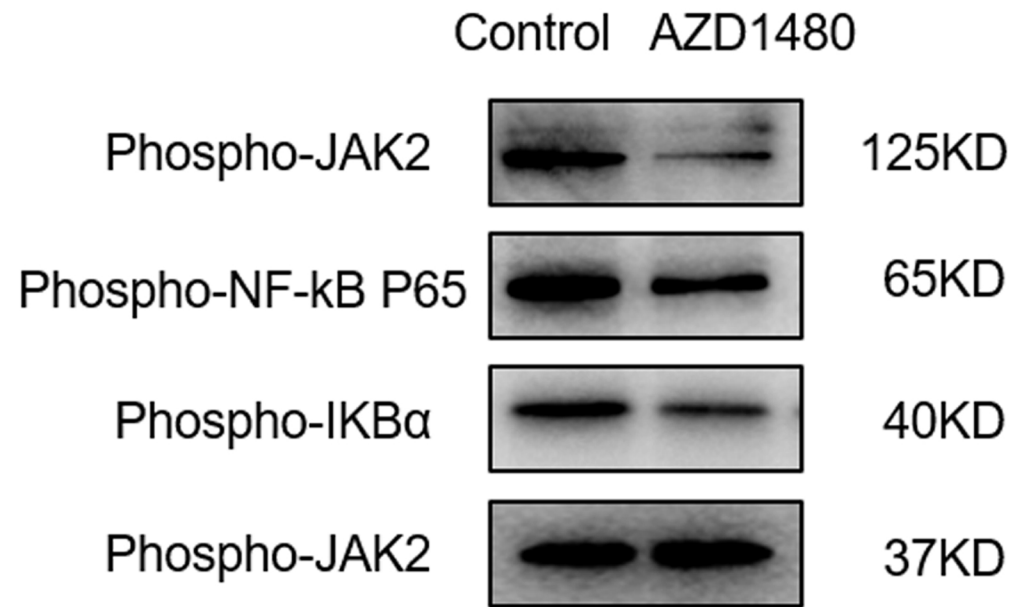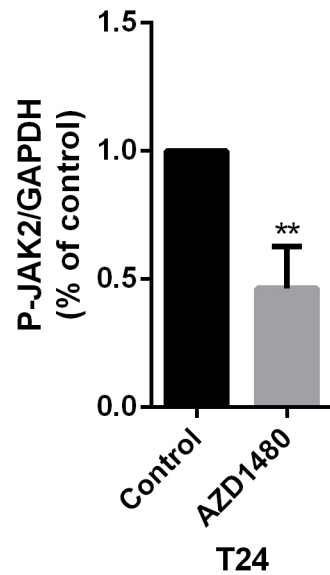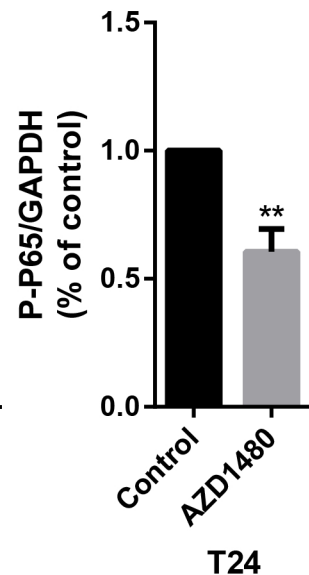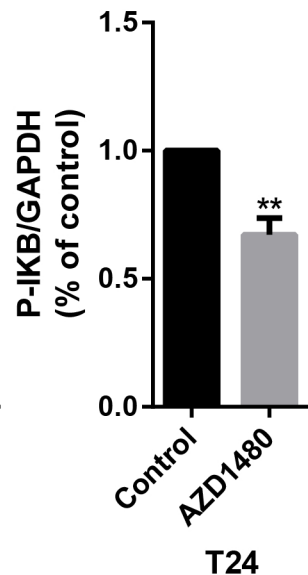

Supplement: Supplementary file 1 — Additional file 1: Supplementary Figure 1. The effect of JAK2 inhibitor (AZD1480) on T24 cells. p-JAK2, p-P65, and p-IKB were downregulated when T24 cells were treated with AZD1480. *P < 0.05, **P < 0.01 compared with the control group(n = 3). [file 12906_2021_3318_MOESM1_ESM.pdf]

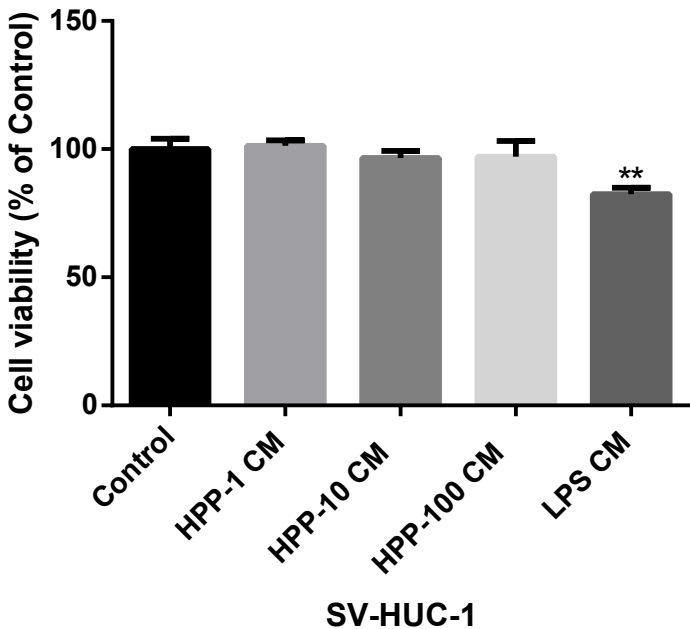

Supplement: Supplementary file 2 — Additional file 2: Supplementary Figure 2. The effect of HPP-treated macrophage on normal bladder epithelial cells (SV-HUC-1). The conditioned mediums from HPP-treated macrophage have no effect on normal cells while LPS-activated macrophage is not. *P < 0.05, **P < 0.01 compared with the control group(n = 3). [file 12906_2021_3318_MOESM2_ESM.pdf]

**A**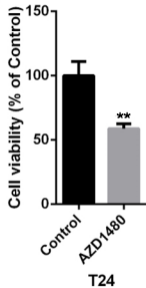**B**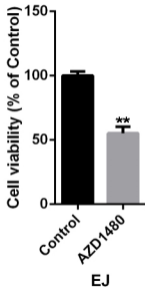**C**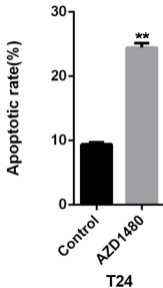**D**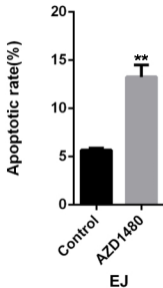

Supplement: Supplementary file 3 — Additional file 3: Supplementary Figure 3. The effect of JAK2 inhibitor (AZD1480) on bladder cancer cells. AZD1480 inhibited the cell viability and induced apoptosis of bladder cancer cells. *P < 0.05, **P < 0.01 compared with the control group(n = 3). [file 12906_2021_3318_MOESM3_ESM.pdf]
